# IL-33 induces granzyme C expression in murine mast cells via an MSK1/2-CREB-dependent pathway

**DOI:** 10.1042/BSR20221165

**Published:** 2022-12-06

**Authors:** Iain R. Phair, Megan C. Sumoreeah, Niamh Scott, Laura Spinelli, J. Simon C. Arthur

**Affiliations:** Division of Cell Signalling and Immunology, School of Life Sciences, University of Dundee, Dundee DD1 5EH, U.K.

**Keywords:** CREB, extracellular signal-regulated kinases, interleukins, mast cell, Myd88, p38 MAPK

## Abstract

Granzymes comprise a group of proteases involved in the killing of infected or cancerous cells by the immune system. Although best studied in T cells and natural killer (NK) cells, they are also expressed in some innate immune cells. Granzymes B and C are encoded in the mouse chymase locus that also encodes a number of mast cell-specific proteases. In line with this, mast cells can express granzyme B, although how this is regulated and their ability to express other granzymes is less well studied. We therefore examined how IL-33, a cytokine able to activate mast cells but not induce degranulation, regulated granzyme B and C levels in mast cells. Granzyme C, but not B, mRNA was strongly up-regulated in bone marrow-derived mast cells following IL-33 stimulation and there was a corresponding increase in granzyme C protein. These increases in both granzyme C mRNA and protein were blocked by a combination of the p38α/β MAPK inhibitor VX745 and the MEK1/2 inhibitor PD184352, which blocks the activation of ERK1/2. ERK1/2 and p38α activate the downstream kinases, mitogen and stress-activated kinases (MSK) 1 and 2, and IL-33 stimulated the phosphorylation of MSK1 and its substrate CREB in an ERK1/2 and p38-dependent manner. The promoter for granzyme C contains a potential CREB-binding site. Bone marrow-derived mast cells from either MSK1/2 double knockout or CREB Ser133Ala knockin mice were unable to up-regulate granzyme C. Together these results indicate that IL-33-induced granzyme C expression in mast cells is regulated by an MSK1/2-CREB-dependent pathway.

## Introduction

Mast cells are tissue resident granulocytes that are most abundant at the interface between tissues and the external environment, such as around blood vessels, in the skin or mucosal surfaces in the lungs and gut (reviewed in [1–3]). Pathologically, they are involved in allergic reactions and anaphylaxis; however, they may also play protective roles in responses to some infections, particularly to pathogenic helminths [4,5]. Mast cells are characterised by the presence of cytoplasmic secretory granules that contain a number of preformed inflammatory mediators, including proteases and histamine [3]. A number of different stimuli can lead to mast cell degranulation and rapid release of these inflammatory mediators including stimulation of the IgE receptor, complement proteins C3a and C5a, and some peptides including the neuropeptide substance P and peptides derived from venoms [4–6]. In addition to degranulation, mast cells can also be stimulated to secrete cytokines in the absence of degranulation via the activation of Toll-like receptors. For example, both human and murine mast cells have been found to produce cytokines in response to the TLR4 agonist LPS [7,8]. Mast cells also express high levels of the IL-33 receptor [9], which like TLRs, activates Myd88-dependent signalling pathways to drive *de novo* cytokine production in mast cells [10].

A number of different proteases are present in the granules of mast cells, including mast cell-specific tryptases and chymases, the metalloprotease Carboxypeptidase A3, cathepsins, and granzyme B [3]. Both the mast cell chymase and granzyme B genes are located at the same chromosomal locus. This locus has undergone expansion during mammalian evolution and as a result the number of genes present varies across different mammalian species [11]. The human genome contains a single chymase gene, *Cma1*, followed by cathepsin G, granzyme H, and the granzyme B. In contrast, in mice, more genes are present in this locus with the *Cma1* gene, followed by additional chymase genes (*Mpct1*, *9*, *2*, *4*, and then *8*) before the cathepsin G gene (*Ctsg*) [12]. While granzyme B is conserved between mice and humans, mice do not have a direct equivalent of granzyme H but instead the locus is expanded to contain genes for granzymes E, D, G, N, F, and C [12].

Granzymes have attracted most attention for their role in T-cell and natural killer (NK)-cell function, and multiple studies have addressed how the expression of specific granzymes is regulated in T cells (reviewed in [13–15]). While it has been shown that mast cells express granzymes [16–18], less is known about how their expression is regulated in these cells. Granzyme B expression has been reported in mature mast cells in the skin in both humans and mice as well as in human cord blood-derived mast cells and mouse bone marrow-derived mast cells [17,18]. Furthermore, mRNA expression for granzyme B can be up-regulated by PMA/ionomycin treatment in both human cord blood-derived mast cells and the LAD2 mast cell line. Granzyme H has been found in cord blood-derived mast cells, and its mRNA is up-regulated in these cells in response to ionomycin treatment or FcεR cross-linking [16]. Granzyme D mRNA has also been detected in murine bone marrow-derived mast cells, although the levels were over 1000 times lower than for Granzyme B [19].

IL-33 is a member of the IL-1 family known to stimulate a number of immune cell types including mast cells (reviewed in [20–22]). IL-33 is a strong activator of *de novo* cytokine production in mast cells without inducing degranulation, although it has also been shown to synergise with other signals to promote degranulation. We show here that IL-33 up-regulates granzyme C, but not granzyme B or Cma1 protein expression in bone marrow-derived mast cells and that this is regulated via a mitogen and stress-activated kinases (MSK) 1/2-CREB-dependent pathway.

## Methods

### Mice

The generation of MSK1/2 double knockout and conditional CREB S133A knockin mice have been described previously [23–25]. To generate the CREB S133A knockin mutation specifically in haemopoietic cells the CREB mice were crossed to a Vav-iCre transgene [26]. All lines were backcrossed to C57Bl6/J (Charles River Laboratories, U.K.) for at least 12 generations. Animals were maintained in individually ventilated cages under specific pathogen-free conditions and housed in line with UK and EU regulations. Nonbreeding mice were kept in same sex groups and allowed free access to water and food (R&M1 SDS, Special Diet Services). Animal rooms were maintained on a 12/12-h light/dark cycle at 21°C and between 45 and 65% humidity. Mice were sacrificed using a rising concentration of CO_2_ in their home cage and death confirmed by cervical dislocation. All work were carried at the University of Dundee out under a UK Home Office licence (PAAE38C7B) and approved by the University of Dundee Ethical Review and Welfare Committee.

### Culture of bone marrow-derived mast cells

Bone marrow-derived mast cells (BMMCs) were cultured as described previously [27]. Briefly, bone marrow was flushed in PBS and the cells pelleted by centrifugation. Cells were cultured at 1 million cells per ml in RPMI 1640 supplemented with 10% FBS (Biosera/Labtech), 5 mM l‐Glutamine (GIBCO Life Technologies), 100 μg/ml Penicillin (GIBCO Life Technologies), 100 μg/ml streptomycin (GIBCO Life Technologies), 25 mM HEPES (Lonza), 1 mM sodium pyruvate (Lonza), 1× nonessential amino acids (Lonza), 50 μM 2‐mercaptoethanol, and 30 ng/ml IL‐3 (PeproTech). Cells were passaged twice per week and used between passages 12 and 16. Where indicated, BMMCs were treated for 1 h with 2 µM of the MEK1/2 inhibitor PD184352 or 1 µM of the p38α/β inhibitor VX745 before stimulation with 10 ng/ml IL-33. The concentrations of the inhibitors were selected as they are the minimum concentration required to fully inhibit their targets based on the previous studies [28,29].

### qPCR

Analysis of mRNA induction was carried out as described previously [30]. Briefly, total RNA was isolated using NucleoSpin RNA Plus mini isolation kits (Macherey-Nagel) according to the manufacturer’s protocols. RNA was reverse transcribed using iScript (BioRad) and qPCR carried out as described using SYBR green-based detection methods. The levels of granzyme mRNA were quantified relative to GAPDH mRNA levels using the equation:

$$\text{relative mRNA level}\;=2^{\left(\text{Ct}(\text{GAPDH})-\text{Ct}(\text{granzyme})\right)}\;$$

Similar results were obtained if granzyme was normalised to 18s levels (not shown). Primer sequences are shown in Table 1.

**Table 1:** Primer sequences used for qPCR

mRNA	Forward	Reverse
GAPDH	GTAACCCGTTGAACCCCATT	CCATCCAATCGGTAGTAGCG
18S	ACAGTTCTTATGTGGTGACCC	TGCACCACCAACTGCTTAG
Granzyme C	CATCGTCTCCTACGGGCAAA	CCTGGACTCAGCTATGGGGA
Granzyme B	GAAGCCAGGAGATGTGTGCT	GCACGTTTGGTCTTTGGGTC

### Proteomics sample preparation

Four independent BMMC cultures were either stimulated with 10 ng/ml IL-33 for 48 h or left unstimulated. Cells were then centrifuged at 340 *g* for 5 min at 4°C, resuspended in Hanks’ balanced salt solution (HBSS; Gibco) and transferred to 2 ml Protein Lo-Bind microcentrifuge tubes (Eppendorf). Cells were then washed twice in HBSS by pelleting with centrifugation at 9000 *g*, 4°C for 20 s. Cells were resuspended in 400 µl lysis buffer (4% SDS, 10 mM tris(2-carboxyethyl)phosphine (TCEP) and 50 mM triethylammonium bicarbonate (TEAB), pH 8.5) and incubated for 5 min at room temperature with shaking. Samples were then heated for 5 min at 95°C with shaking on a ThermoMixer, followed by sonication with a BioRuptor (15 cycles of 30 s on/30 s off). Protein concentration was determined using the EZQ Protein Quantitation Kit (Invitrogen) as per manufacturer’s instructions. Samples were alkylated with 20 mM iodoacetamide (IAA) in the dark at room temperature for 1 h. A total of 200 µg SP3 beads were added to each sample, followed by 550 µl of 90% acetonitrile:10% formic acid and samples incubation for 8 min at room temperature with shaking. Samples were placed on a magnetic rack and incubated for 2 min to facilitate binding of the beads. Supernatant was removed, and beads were washed twice in 70% EtOH, and once in 100% acetonitrile. Beads were then air dried for 15 s before being resuspended in 65 µl 50 mM ammonium bicarbonate. Lys-C was added to samples at a 1:100 ratio, and samples were incubated overnight at 37°C with gentle agitation. Trypsin was then added to samples at a 1:100 ratio, and samples were incubated overnight at 37°C with gentle agitation. Beads were then resuspended in 1425 µl acetonitrile and incubated for 8 min at room temperature with shaking. Samples were then placed on a magnetic rack and incubated for 2 min. Supernatant was removed, beads were washed once in 500 µl acetonitrile and beads were air dried for 15 s. A total of 189 µl 2% DMSO was added to each sample, followed by sonication with a BioRuptor (five cycles of 30 s on/30 s off). Samples were centrifuged, placed on a magnetic rack, and incubated for 2 min before transferring the supernatant containing peptides to fresh 1.5 ml Protein Lo-Bind microcentrifuge tubes. Samples were then clarified by centrifugation at 20000 *g* for 10 min, followed by addition of formic acid to give a final concentration of 5% formic acid. Peptide quantitation was performed using a CBQCA assay (Thermo Fisher Scientific).

### Single-shot LC-MS analysis

Analysis of peptides was performed on a Q-exactive HFX mass spectrometer (Thermo Scientific) coupled with a Dionex Ultimate 3000 RS (Thermo Fisher Scientific). The following LC buffers were used: buffer A (0.1% formic acid in Milli-Q water (v/v)) and aliquots of 15 µl of each sample were loaded at 10 µl/min onto a trap column (100 µm, 2 cm, Acclaim PepMap nanoViper C18 column, 5 µm, 100 Å, Thermo Fisher Scientific) in loading buffer (0.1% formic acid). The trap column was washed for 5 min at the same flow rate, then the trap column was switched in-line with a resolving C18 column (75 µm, 50 cm, Acclaim PepMap RSLC C18 column, 2 µm, 100 Å, Thermo Fisher Scientific). The peptides were eluted from the column at a constant flow rate of 300 nl/min with a linear gradient from 5 to 35% HPLC buffer (80% acetonitrile and 0.08% formic acid in Milli-Q water (v/v)) in 120 min, and then to 98% HPLC buffer by 122 min. The column was then washed with 98% HPLC buffer for 15 min and re-equilibrated in 2% HPLC buffer for 17 min. The Q-exactive HFX was used in data-dependent mode. A scan cycle comprised MS1 scan (m/z range from 335 to 1800, with a maximum ion injection time of 50 ms, a resolution of 60000 and AGC value of 3 × 10^6^), followed by 40 sequential dependant MS2 scans (with an isolation window set to 1.4 Da, resolution at 7500, maximum ion injection time at 50 ms, and AGC value of 1 × 10^5^).

### Database searching and reporter ion quantification

The data were processed, searched, and quantified with the MaxQuant software package (version 1.6.10.43). Proteins and peptides were identified using the Uniprot mouse reference proteome database released 2020 06 (SwissProt and Trembl) and the contaminants database integrated in MaxQuant using the andromeda search engine [31–33] with the following search parameters: Trypsin and LysC were selected as the proteolytic enzymes with a maximum of two missed cleavages permitted, carbamidomethylation of cysteine was set as a fixed modification, and the following variable modifications were selected: oxidation of methionine, glutamine to pyroglutamate, deamidation of asparagine and glutamine, and acetylation of the protein N-terminus. The false discovery rate was set to 1% for positive identification of proteins and peptides with the help of the reversed mouse Uniprot database in a decoy approach. Estimates of copy numbers and cellular protein concentration were calculated using the histone ruler method [34]. The mass spectrometry proteomics data have been deposited to the ProteomeXchange Consortium via the PRIDE [35] partner repository with the dataset identifier PXD033759. The processed data are shown in Supplementary Table S1.

### Flow cytometry

Cell staining was done on ice in the dark, and centrifugation steps were performed at 340 *g*, 4°C for 5 min. Cells were pelleted by centrifugation, resuspended in PBS with 1% BSA, and incubated with Fc Block (BD Biosciences; 1:50) for 10 min. Cells were fixed and permeabilised using the Fix-Perm Kit (BD Biosciences) according to the manufacturer’s protocol. The cells were then incubated with the granzyme C antibody (clone SCF1D8 labelled with PE, Biolegend) for 30 min in the dark at 4°C washed twice, and acquired on a FACSCanto II. Data were analysed using FlowJo software.

### Immunoblotting

Two million BMMCs were pelleted by centrifugation and lysed in SDS lysis buffer (40 mm Tris‐HCl (pH 7.5), 0.8 mm EGTA, 0.8 mm EDTA, 0.8 mm sodium orthovanadate, 40 mm sodium fluoride, 0.8 mm sodium pyrophosphate, 0.22 m sucrose, 1% (v/v) Triton X‐100, 0.1% (v/v) 2‐mercaptoethanol, 2% (w/v) SDS, and 10% glycerol). Lysates were heated at 95°C for 5 min and DNA sheared by passing the lysates through a 25-gauge needle. Samples were run on 10% SDS tris/glycine polyacrylamide gels and transferred on to nitrocellulose membranes using standard techniques. Primary antibodies were from Cell Signalling Technologies. Horse radish peroxidase-conjugated secondary antibodies were from Thermo Fisher Scientific. Detection was carried out using Clarity ECL reagent (Bio-Rad) and imaged on an Odyssey Fc system (Licor) and analysed using Image Studio software. Full membrane images of the blots are shown in Supplementary Figure S1.

### Statistical analysis

Data were analysed using GraphPad Prism. Group sizes and the tests used are detailed in the figure legends. For the proteomic data sets in Supplementary Figure S2, data were log transformed before calculation of *P-*values using unpaired two tailed tests. False discovery rate (FDR) was then performed using the two-stage step up method of Benjamini, Krieger, and Yekutieli using a Q value of 5%.

## Results

### IL-33 induces granzyme C expression in BMMCs

**Figure 1:**
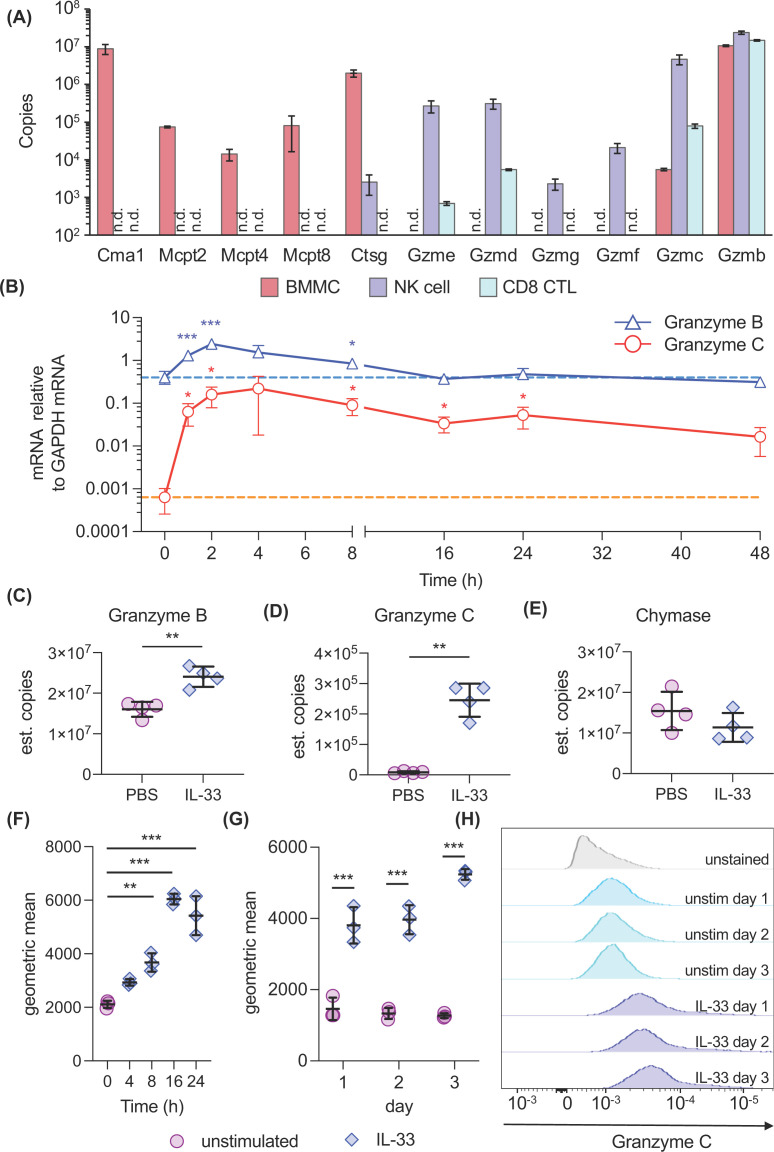
IL-33 induces granzyme c expression in BMMCs (**A**) The average copy numbers of proteins in the mouse chymase locus detected in BMMCs, NK cells, and CD8 cytotoxic T (CTL) cells proteomes in the ImmPres database (http://immpres.co.uk/). Gene abbreviations are: Cma1, Chymase; Mcpt, Mast Cell protease; Ctsg, Cathepsin G; Gzm, Granzyme. (**B**) BMMCs were stimulated with 10 ng/ml IL-33 for the indicated times. Cells were then lysed and total RNA isolated. The levels of granzyme B and granzyme C were determined by qPCR and normalised to the level of GAPDH mRNA in the same sample. Data show mean and 95% confidence interval of four stimulations. For comparisons to the 0 h time point, a *P*<0.05 is indicated by * and *P*<0.001 by *** (Brown-Forsythe and Welch ANOVA and Dunnett’s post hoc testing). (**C–E**) BMMCs were stimulated with 10 ng/ml IL-33 for 48 h and lysates generated for proteomics analysis as described in the Methods section and the estimated copy numbers of granzyme B (**C**), granzyme C (**D**), and chymase (**E**) determined. Graphs show mean and standard deviation of four independent cultures. A *P*<0.05 is indicated by * and *P*<0.01 by ** (two-tailed unpaired ttest with Welch’s correction). (**F–H**) BMMCs were incubated with 10 ng/ml IL-33 for the indicated times or maintained in the absence of IL-33 (unstimulated) as indicated. Cells were then fixed, stained for granzyme C, and analysed by flow cytometry as described in the Methods section. A short time course of IL-33 stimulation is shown in (**F**), while a separate experiment looking at IL-33 treatment for 1–3 days is shown in (**G**), with representative flow cytometry histograms in (**H**). The graphs show the mean and standard deviation of three separate stimulations. For comparisons to the unstimulated cells, a *P*<0.01 is indicated by ** and *P*<0.001 by ***. Data in (**F**) were analysed by one-way ANOVA and Dunnett’s post hoc testing and (**G**) was analysed by two-way ANOVA with Sidak’s post hoc testing between unstimulated and IL-33-stimulated conditions.

Analysis of mass spectrometry data available in the ImmPres (http://immpres.co.uk) data base indicated that of the genes in the chymase locus, BMMCs expressed chymase, cathepsin G, and granzyme B proteins, along with lower levels of granzyme C, Mcpt2, Mcpt4, and Mcpt8 (Figure 1A). Of the other granzymes encoded in this locus, granzymes E, D, G, and F were not detected in BMMCs but were found in NK cells, another cell type known to contain high levels of granzymes (Figure 1A). Granzyme N was not detected in either BMMCs or NK cells in these experiments. To further examine the expression of granzymes B and C in BMMCs, cells were stimulated with IL-33, and RNA isolated and analysed by qPCR. This showed that, consistent with the higher protein level of granzyme B relative to granzyme C in BMMCs, the mRNA level for granzyme B was 600-fold higher than granzyme C in unstimulated cells. IL-33 had little effect on the mRNA level of granzyme B, with a maximal induction of sevenfold and a return to basal levels by 16 h (Figure 1B). In contrast, IL-33 resulted in a large increase in granzyme C mRNA, with the maximal induction of approximately 350-fold seen around 2 h after IL-33 stimulation (Figure 1B). While this decreased over time, granzyme C levels were still increased by approximately 25-fold 38 h after the IL-33 stimulation. To examine if granzyme protein levels changed, lysates from control or IL-33-stimulated cells were analysed via single-shot DDA mass spectrometry as described in the Methods section. A total of 2992 proteins were identified in at least three of the four replicates in each condition (Supplementary Table S1 and Supplementary Figure S2A), including granzymes B and C. Protein copy numbers and concentrations were estimated using the histone ruler method and this indicated that IL-33 resulted in a small increase in the predicted protein mass inside the cell, suggesting an increase in cell volume (Supplementary Figure S2B). Consistent with a change in cell size, there was an increase in forward and side scatter, following IL-33 stimulation when mast cells were analysed by flow cytometry (Supplementary Figure S2C). Using cutoff of a twofold change and an FDR Q value of 5%, the copy number of 19 proteins was decreased and 132 were increased, including granzyme C (Supplementary Figure S2D and Supplementary Table S2). If estimated concentrations were used to account for the change in total protein content, 36 proteins were decreased, and 39 were increased using the same cutoff values (Supplementary Figure S2E and Supplementary Table S2). A small increase (1.5-fold) was observed in the estimated protein copy number of granzyme B (Figure 1C). This was similar to the increase in protein content and analysis of the change in the estimated protein concentration of granzyme B showed little effect of IL-33 (Supplementary Figure S2F). In contrast, granzyme C protein copy numbers were strongly increased after 48 h of IL-33 stimulation (Figure 1D), and this increase was also apparent if estimated concentrations were analysed (Supplementary Figure S2F). Chymase was also detected; however, its copy number was not increased following IL-33 stimulation (Figure 1E). To confirm and extend these findings on granzyme C expression, BMMCs were fixed and granzyme C expression was analysed by intracellular staining and flow cytometry. Granzyme C expression gradually increased over the first 16 h of IL-33 stimulation before starting to plateau (Figure 1F). The increase in granzyme C levels seen at 24 h was maintained at 48 and 72 h (Figure 1G,H).

**Figure 2:**
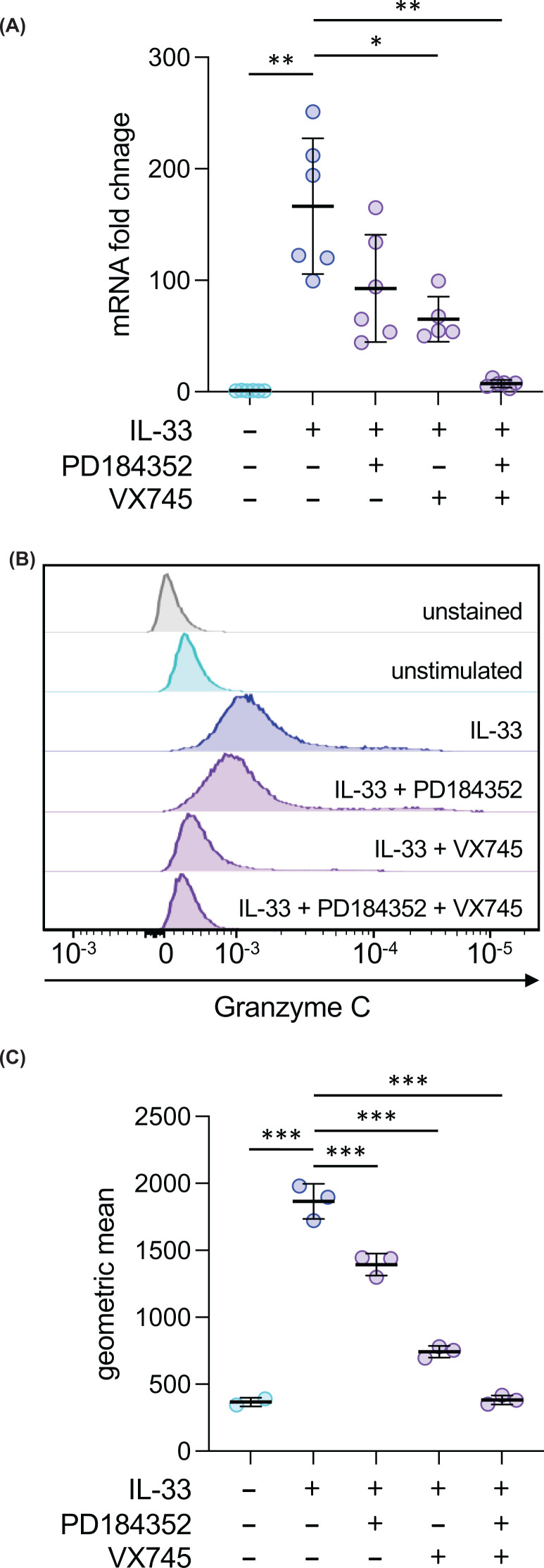
Granzyme C induction requires ERK1/2 and p38 MAPK signalling (**A**) BMMCs were incubated with either 2 μM PD184352 or 1 μM VX745 as indicated for 1 h before stimulation with 10 ng/ml IL-33 for a further 1 h. Total RNA was isolated and the induction of granzyme C mRNA determined by qPCR. For comparisons with the IL-33 only condition, a *P*<0.05 is indicated by *, and *P*<0.01 by ** (Brown-Forsythe and Welch ANOVA followed Dunnett’s T3 post hoc testing). (**B,C**) BMMCs were incubated with either 2 μM PD184352 or 1 μM VX745 as indicated for 1 h before stimulation with 10 ng/ml IL-33 for 24 h. Cells were then fixed, stained for granzyme C, and analysed by flow cytometry. Representative histograms are shown in (**B**) and quantification of the geometric mean in (**C**). Graphs show the mean and standard deviation of three independent stimulations. For comparisons to the IL-33 only condition, a *P*<0.05 is indicated by *, *P*<0.01 by ** and *P*<0.001 by *** (one-way ANOVA followed Dunnett’s T3 post hoc testing).

Previously, it has been shown that IL-33-induced cytokine responses in mast cells are dependent on the activation of the p38 MAPK pathway [27,36,37]. To determine if MAPK signalling was also required for granzyme C induction, cells were treated with either the p38α/β inhibitor VX745 [38] or the MEK1/2 inhibitor PD184352, which blocks the activation of the ERK1/2 MAPKs [28], before stimulation with IL-33. Both VX745 and PD184352 caused a reduction in the induction of granzyme C mRNA, with a combination of both VX745 and PD184352 having a greater effect than either inhibitor alone (Figure 2A). In line with the mRNA data, both VX745 and PD184352 caused a partial reduction in granzyme C protein levels in IL-33-stimulated BMMCs, with a combination of VX745 and PD184352 completely blocking the IL-33-stimulated increase in granzyme C protein (Figure 2B,C).

### Granzyme C is regulated by MSK1/2 and CREB

**Figure 3:**
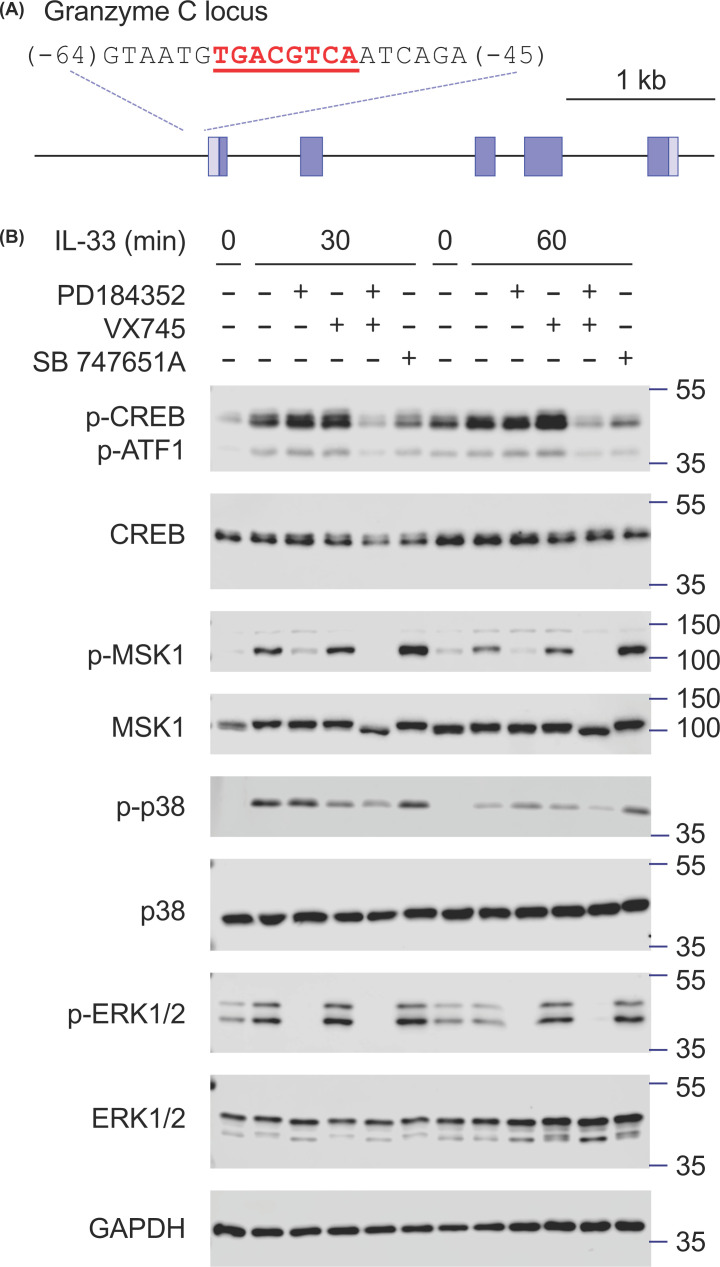
IL-33 induces MSK1 activation and phosphorylation of CREB (**A**) Schematic of the murine granzyme C locus showing the five exons. A consensus-binding site for CREB (underlined text) is located 54bp of the transcriptional start site annotated in Ensembl (transcript ENSMUST00000015585.4). (**B**) Wild-type BMMCs were incubated with either 2 μM PD184352 or 1 μM VX745 or 10 μM SB 747651A as indicated for 1 h before stimulation with 10 ng/ml IL-33 for 30 min. The levels of phospho-ERK1/2, total ERK1/2, phopho-p38, total p38, phospho-MSK1, total MSK1 phospho-CREB, total CREB, and GAPDH were determined by immunoblotting.

Both p38α/β and ERK1/2 can activate the MSK1 and MSK2, which are known to regulate the transcription of target genes via the phosphorylation of CREB on Ser133 [23,39]. Interestingly, the mouse granzyme C promoter contains a consensus CREB-binding site, suggesting that MSKs may be involved in regulating granzyme C transcription downstream of IL-33 (Figure 3A). In other systems, it has been shown that combined inhibition of both ERK1/2 and p38α is required to fully inhibit MSK activation [40]. Consistent with the previous reports [27,36,37], IL-33 induced ERK1/2 and p38α activation in BMMCs, as judged via phosphorylation on their T-X-Y activation motifs (Figure 3B). MSK1 was also activated, as judged both by the phosphorylation of MSK1 on T581 as well as the phosphorylation of the MSK substrate CREB on Ser133 (Figure 3B). PD184352 alone was able to reduce MSK1 phosphorylation while VX745 on its own had little effect. A combination of PD184352 and VX745 was however required to fully block MSK1 phosphorylation (Figure 3B). MSK1 is phosphorylated on multiple sites upon activation, resulting in a band shift on SDS gels that correlates with the full activation of MSK1 (Figure 3B). A combination of both PD184352 and VX745 was required to prevent this band shift (Figure 3B, compare lanes 2/7 with 5/11 on the total MSK1 blot). In line with this, a combination of PD184352 and VX745 was required to fully block CREB phosphorylation in response to IL-33. CREB phosphorylation was also blocked by the MSK1/2 inhibitor SB 747651A (Figure 3B, a compound that inhibits the N-terminal kinase domain of MSK1 but does not affect its phosphorylation by the upstream MAPKs) [39].

**Figure 4:**
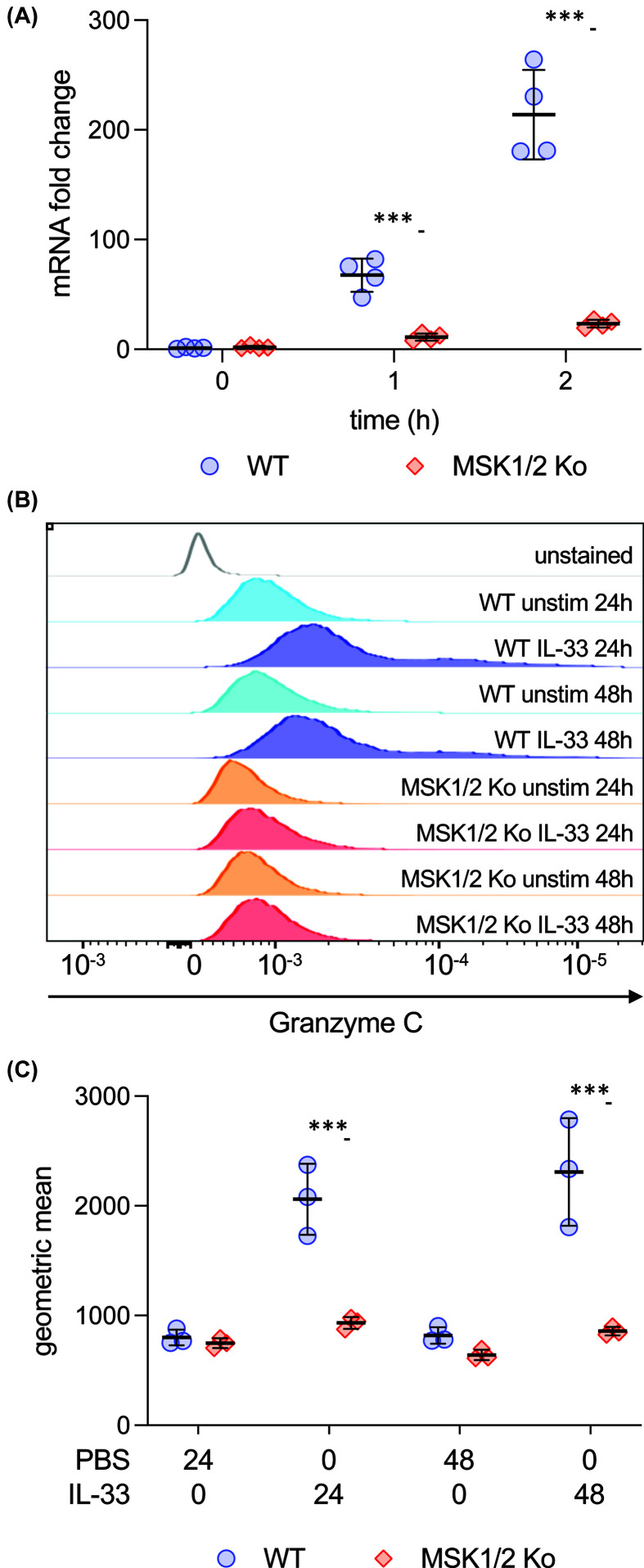
MSK1 and 2 are required for granzyme C induction in BMMCs (**A**) Independent cultures of BMMCs from four wild-type and four MSK1/2 mice were isolated and stimulated with 10 ng/ml IL-33 for 0, 1, or 2 h. Total RNA was isolated and the induction of granzyme C mRNA determined. Fold-change was calculated relative to the level in unstimulated wild-type BMMCs. An average of GAPDH and 18s levels were used to correct for loading. (**B,C**) Wild-type and MSK1/2 knockout BMDMs were stimulated with 10 ng/ml IL-33 for 24 or 48 h and the levels of intracellular granzyme C determined by flow cytometry. Representative plots are shown in (**B**) and quantification of the geometric mean from three mice per genotype in (**C**). For (**A**) and (**C**), graphs show the mean and standard deviation with individual cultures shown as symbols. For comparisons with the IL-33 only condition, a *P*<0.001 is indicated by *** (two-way ANOVA followed Sidak’s post hoc testing).

MSK1 and 2 are closely related kinases and previous studies have shown that they can compensate for each other, and double knockout of MSK1 and 2 is required to fully block the phosphorylation of MSK substrates [23,41]. To determine if MSKs were required for the induction of granzyme C, BMMCs were generated from MSK1/2 double knockout mice. Compared with wild-type cells, MSK1/2 knockout cells showed considerably lower induction of granzyme C mRNA in response to IL-33 stimulation (Figure 4A). To confirm a similar effect was also seen at the protein level, granzyme C induction was determined at 24 and 48 h after IL-33 stimulation by flow cytometry. This showed that unlike wild-type cells, MSK1/2 knockout BMMCs were unable to induce granzyme C in response to IL-33 (Figure 4B,C).

**Figure 5:**
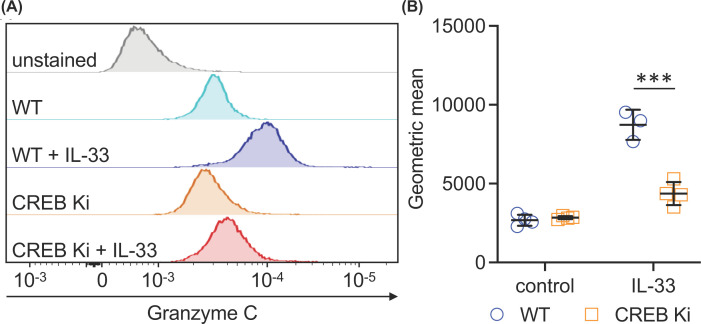
CREB phosphorylation regulates granzyme C induction BMMCs from three wild-type or four CREB Ser133Ala mice were stimulated with 10 ng/ml IL-33 for 24 h or left unstimulated. The levels of intracellular granzyme C were then determined by flow cytometry. Representative histograms are shown in (**A**) and quantification of the geometric mean in (**B**). The graph shows the mean and standard deviation with individual mice shown by symbols. For comparisons to the IL-33 only condition, *P*<0.001 is indicated by *** (two-way ANOVA followed Sidak’s post hoc testing).

To confirm that MSKs regulated granzyme C via CREB and not an alternative substrate, BMMCs were generated from mice with a conditional knockin mutation to Ser133 to alanine in the endogenous CREB gene. In these mice, the Ser133Ala knockin mutation was generated in the endogenous CREB gene and a floxed wild-type CREB minigene placed upstream to allow expression of wild-type CREB. Crossing these mice to Vav-iCre transgenic line resulted in mice where the minigene was excised in haemopoietic cells, resulting in expression of the CREB Ser133Ala mutant in these cells. Similar to the MSK1/2 knockout BMMCs, the CREB Ser133Ala knockin BMMCs did not up-regulate granzyme C to the same extent as wild-type cells in response to IL-33 (Figure 5).

## Conclusion

We show here that IL-33 can induce granzyme C protein expression in BMMCs but that granzyme B expression is largely unaffected. While the genes granzyme B and C are localised to the same region of chromosome 14 in mice, their expression has previously been found to be independently regulated in T cells [42,43]. The induction of granzyme C required the activation of MSK1/2 and phosphorylation of CREB downstream of the ERK1/2 and p38 MAPK pathways. Interestingly, inhibition of p38 alone had little effect of CREB phosphorylation; however, it did result in a partial reduction in granzyme C induction. This could suggest additional roles for p38 in addition to activating MSK1 and 2. One possible mechanism for this could be via the activation of the kinases MK2 and 3 downstream of p38α. In addition to granzyme C, IL-33 is known to promote the production of cytokines including TNF, IL-6, IL-13, and GM-CSF in BMMCs, and MK2 and 3 have been found to be critical for this process [27,36,37]. This p38α-MK2/3 pathway also regulates IL-33-induced cytokine production in dendritic cells [44] and type 2 innate lymphoid cells [45] and is also important downstream of TLR activation in multiple innate immune cells (reviewed in [46]). In contrast, loss of MSK1 and 2 does not affect IL-33-induced cytokine production in BMMCs [27]. MSK1/2 activation has also been reported downstream of TLR agonists. In macrophages, MSKs phosphorylate CREB and thereby promote the transcription of the anti-inflammatory cytokine IL-10 and MSK1/2 knockout mice are sensitised to LPS-induced endotoxic shock [47]. The roles of MSK1/2 do however appear to be cell-type dependent as in contrast with macrophages MSK1 and 2 are not required for IL-10 induction in LPS-stimulated B cells [48]. Granzyme C does not possess a direct homologue in human cells; however, granzyme H has been suggested to be the closest human granzyme to murine granzyme C and, similar to granzyme C, is encoded in the chymase locus [12,49]. Alignment of the human granzyme H and mouse granzyme C promoter regions shows the CRE site in the mouse promoter is not conserved in human granzyme H promoter. Further analysis of the 3kb proximal promoter sequence of granzyme H does not reveal a full CRE consensus sequence (TGACGTCA). Although there is a potential half-site (TGACG); however, it is not clear if this site is functional *in vivo* (Supplementary Figure S3). Further work would be required to determine how granzyme H transcription is regulated.

The role of granzymes produced by mast cells requires further investigation. Granzyme B is able to induce apoptosis in cancer cell or cells infected with viral or bacterial pathogens via stimulating apoptosis. Granzyme C is also reported to promote apoptosis in cells, although this occurs via mitochondrial swelling and is caspase independent [50]. In order to stimulate apoptosis, the entry of granzyme into the target cells requires the pore-forming perforin [13–15]. In contrast with other cells, such as T cells and NK cells that express high levels of granzymes, mast cells do not appear to express perforin [17]. Thus, the role of mast cell granzymes would appear to be perforin independent. In this respect, very little is known about granzyme C in general or about the role of granzyme B from mast cells. One possibility is that granzymes released from mast cells may promote remodelling of the extracellular matrix. In this respect, it has been shown that granzyme B released from mast cells in culture can cause the detachment and anoikis of murine embryonic fibroblasts in cocultures [17]. A potential role for granzyme B released from mast cells in the regulation of the extracellular matrix *in vivo* is the degradation of the extracellular matrix of tumours, thereby releasing FGF-1 and GM-CSF and promoting angiogenesis [51]. Granzyme B may also play a role in mast cell survival; mast cells are sensitive to lysosomotropic agents. Leakage of granzyme B into the cytosol from vesicles damaged by lysosomotropic drugs is able to promote mast cell apoptosis [52]. Finally granzyme B may also act in a positive feedback loop to sustain IL-33 action; granzyme B released by mast cells has been reported to cleave IL-33 into a more bioactive form [53]. It is possible that granzyme C may play similar roles to granzyme B in mast cells; however, further investigation will be needed to examine this.

In summary, we show here that IL-33 specifically up-regulates granzyme C in BMMCs and this requires that cation of MSK1/2 and the phosphorylation the transcription factor CREB to drive granzyme C mRNA induction.

## Supplementary Material

Supplementary Figures S1-S3

Supplementary Tables S1-S2

## Data Availability

The mass spectrometry proteomics data have been deposited to the ProteomeXchange Consortium via the PRIDE partner repository with the dataset identifier PXD033759.

## Open Access

Open access for the present article was enabled by the participation of University of Dundee in an all-inclusive *Read & Publish* agreement with Portland Press and the Biochemical Society under a transformative agreement with JISC.

## Abbreviations

ANOVA: Analysis of variance
AGC: Automatic gain control
BMMC: Bone marrow-derived mast cell
CRE: Cis-regulatory element 1
CREB: CAMP responsive element binding protein
ERK: Extracellular signal-regulated kinase
EtOH: Ethanol
FBS: Foetal bovine serum
Fc: Crystallizable fragment
FDR: False discovery rate
GAPDH: Glyceraldehyde-3-phosphate dehydrogenase
GM-CSF: Granulocyte-macrophage colony-stimulating factor
HBSS: Hanks’ balanced salt solution
LPS: Lipopolysaccharides
PE: Phycoerythrin
PMA: Phorbol myristate acetate
qPCR: Quantitative PCR
SYBR: SYBR^®^ green RNA stain
TLR: Toll-like receptor
