# Supplementary material for: IL-33 induces granzyme C expression in murine mast cells via an MSK1/2-CREB-dependent pathway
Source: Biosci Rep. 2022 Dec 6;42(12):BSR20221165. doi: 10.1042/BSR20221165 (PMC9727205; doi:10.1042/BSR20221165)
Supplement: Supplementary Figures S1-S3 [file BSR-2022-1165_supp.pdf]

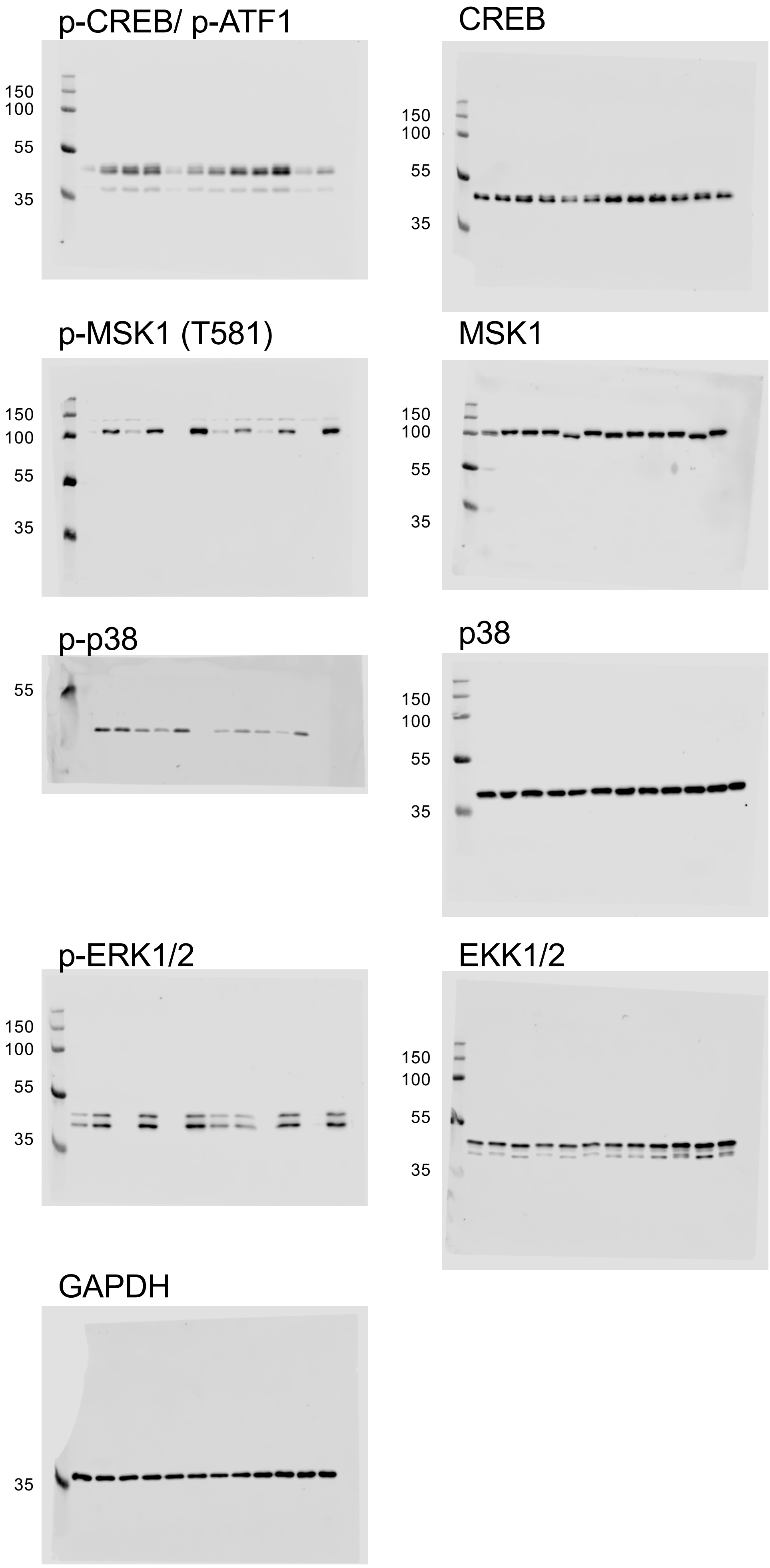

Supplementary figure 1. Immunoblots used for the generation of Figure 3

Immunoblots were run as described in the methods and imaged using an Odyssey Fc system (Licor) and analysed using Image Studio software. Full membrane scans of the blots used to make figure 3 are shown.

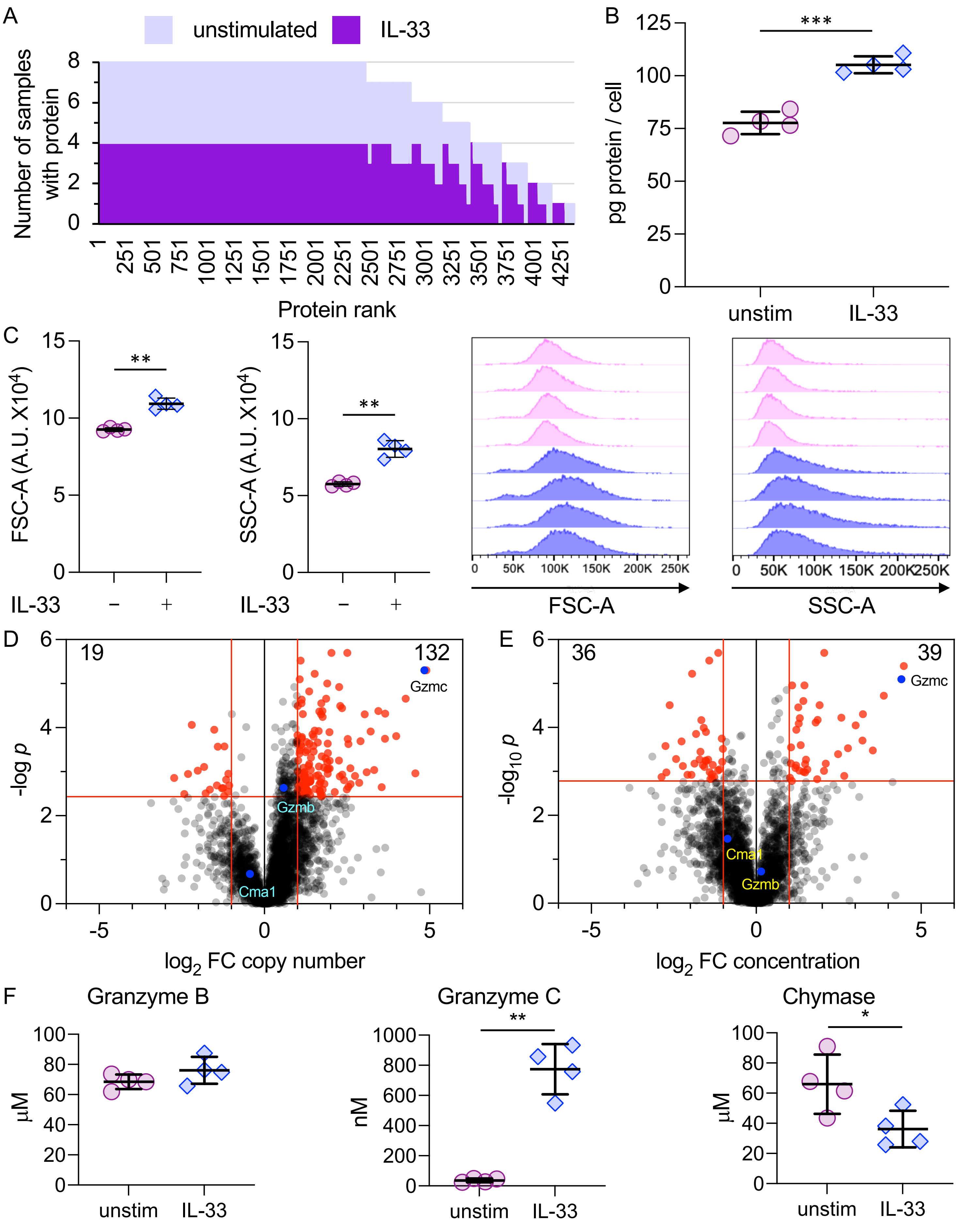

Supplementary figure 2. Proteomic analysis of IL-33 stimulated mast cells

BMMCs were stimulated for 48h with 10ng/ml IL-33 or left unstimulated and then analysed by mass spectrometry as described in the methods. (A) Proteins were ranked in the order of the number of replicates they were detected in (x-axis) and the number of unstimulated and IL-33 treated replicates containing the protein plotted on the y-axis. (B) Total cellular protein estimated for the protein copy number data. (C) Forward (FSC-A) and Side (SSC-A) data from flow cytometry data on unstimulated and 48h IL-33 stimulated BMMCs. (D and E) Volcano plots depicting fold changes in the copy number and concentration data. Red dots indicate proteins meeting a fold change of 2 and FDR q value < 0.05. (F) Estimated concentrations for granzyme B, granzyme C and chymase from the proteomic data. In B, C and F * indicates a p < 0.01, ** p < 0.01 and *** p <0.001 (two tailed unpaired ttest with Welch's correction).

```
mouse_Granzyme_C      aaaataaatattactacttacaggttttcagatatcacaaacatggaccctgctctatta 60
human_Granzyme_H      -aaagtcc----tatcccagttctgttaaagtaatagcaggaatagcaccttataaatac 55
                       ***          * *        **  **  **  **   ** *  ***  *  **

mouse_Granzyme_C      ttttaaaaagattctctacaaatggactc----------------------tcatat--- 95
human_Granzyme_H      ctagaaaaatagtagaaggaaacatgcttaagcattaataatgatctccggtggtagaat 115
                       *  ***** * *      ***    **                       *  **

mouse_Granzyme_C      ---cattaagattattttattctgtaggcttttgttgcttatctgtgtgtgcacatattg 152
human_Granzyme_H      ttttggtaagtttaattttatcatttacatgttcctacttaactatacatatatatttat 175
                            **** *** ***  **  *    * **  * **** ** *   *  * ** *

mouse_Granzyme_C      tgtcatttatgcacagtctctttcagggtgaatgatcaacatttattatagcttgtataa 212
human_Granzyme_H      atatattcatatacataaatatacaaacatatgtatatatacacatttt---------tg 226
                          *** **  ***      * **     *   **  * *   *** *

mouse_Granzyme_C      gcaatgtgcagcctaaaccagtaacctttcaat-------gtagagcagagaagccaaca 265
human_Granzyme_H      tttatggacagcctatgactgtgagccaagggacatcagagacatggctctctggaaact 286
                         ***  *******   * ** * *              *    *       *  ***

mouse_Granzyme_C      tactgcatacccatggactagataattaattttaggcagttagtactttaattgcata 325
human_Granzyme_H      gagagaatacttagtggacagtttttgtttgtttgtttgtttttgagatggagtc 346
                       *  * ****  *  *   ** *  **  ** ** *   *** **  **   ** *  *

mouse_Granzyme_C      aagactaaagactttagtctataactaagatgagaaagcctctcttggttccaaaatat- 384
human_Granzyme_H      tc-gctctgtcacccaggctggagtgcagtggcgcga-tctcagctcactgcaagctcca 404
                          **         ** **  *    **  * *  *  ***   *   * ***  *

mouse_Granzyme_C      ---gacaggagaaaatttaaaagtatctgaaatccataaagcaggataaaaatgtaggtt 441
human_Granzyme_H      cctgggttgacgccattctcctgcctccgcctcccaagtagctggaactacaggagcccg 464
                         *    **    ***     *  ** *    ***   *** ***   * * *

mouse_Granzyme_C      ttggta-gctctgc-agtcttttggatgcatgcgtgcgcgcacacacacacctatgctta 499
human_Granzyme_H      ccaccacgcccggctaatttttttgtatttttagtagagatg--gggtttcaccatgttaa 522
                           * ** * ** * * ***** **   *    * *             * *** * *

mouse_Granzyme_C      gagtgaggtcagccagtctcacctcccagagtaatctcactcagtt---cttactatcta 556
human_Granzyme_H      c---caggatggtctcgatctcctgacctcgtgatccacccgtcttggcctcccaaagtg 579
                           ***   * *    ** ***  *   ** ***   *    **   **  * *  *

mouse_Granzyme_C      ctgttctaaaagaggct-ctcagatcccccagtcaac---atcaaacattt----caaga 608
human_Granzyme_H      ctaggattacaggcatgagccaccgcgcccggccttagtgaacaattattatcaggatga 639
                      **    * * **        **   * *** * *      * ***  ***      * **

mouse_Granzyme_C      cctgcatccttgtctctctccctcttggatgtagcccattaaataacaatcatgatctaa 668
human_Granzyme_H      tcagcatccattttccatcac--------------------------------------- 661
                       * ****** * * **  ** *

mouse_Granzyme_C      taatggactttcttacttgatagaaaatgctaggcaaagtggtagaaaataccattgaga 728
human_Granzyme_H      ----------------ctggcaacaacaactagcatttctcaagggaaatcccctcctca 705
                                       **  *  **   ****      *    * **** ** *    *

mouse_Granzyme_C      gtcaacagcatggaagccagtgctgatctaatcttacctatctttgagttcagtcctgaa 788
human_Granzyme_H      ttcataagcaccatagatggagtggaacagtccccacctcagcttaaggagggctaaata 765
                       ***  ****    **   * *  ** *    *  ****    ** **    *      *

mouse_Granzyme_C      gctcttgcctcagagataaaaggcaatacctagtaaactttaagtgtcattttgatttg 848
human_Granzyme_H      accaaagcata--------aagcaatcagtccactacacttcagtggacccaatgatgac 817
                       *    ** *         ***  *  *       *   ** ****       ****

mouse_Granzyme_C      ttattacacttaactatgtatgcctgtaacacttggtcattggatatggggc----aaac 904
human_Granzyme_H      tcattttggttcaataaatatcaagtccagaactttgtgcagaactgtggaataaaaaa 877
                      * ***    ** * **  ***       * *  *  *     **  ** **     ***

mouse_Granzyme_C      acttgcctt-------taagatttctc------ttgtgaaattttcatagcagtatcagg 951
human_Granzyme_H      agttatctttgtcacagaatatttatatttaaagaggggattctaaatatcccaaaaata 937
                      * **  ***        ** **** *         * * * * *  *** *   *  *

mouse_Granzyme_C      agaatta------gagttaagattaattcatgaggatattcaaagagattgttccacagt 1005
human_Granzyme_H      tgaatctgagatagtggtaagataggaatgggttgccatttgcagtatttgtt---gggt 994
                       ****        * * ******        *  *  ***   **   *****     **

mouse_Granzyme_C      atgatcagtagtcccaaatattaatgagatgatattctgcaatacagtgtgaactctcac 1065
human_Granzyme_H      gtgtgcaactctgcatactgctaaggcaagtccagcctccgtctcac-----accaacag 1049
                       **  **    * *  * *  *** *  *    *  ** *    **      **   **

mouse_Granzyme_C      taagcactgtgtaactgtgagtattataactgctgtaagataaattggttgtcaatattg 1125
human_Granzyme_H      gcagatttccccaccacggcccttaccatcttgtgctctaaccccagattttcagtctac 1109
                        **   *    * *   *    *   * **  **    *      * ** *** * *

mouse_Granzyme_C      ttttaaaaaggaaggaaggaaggaaggaaggaagaaagaaagaaagaaagaaagaa 1185
human_Granzyme_H      tcttgctaaccatcaattcttcaagggcaagagccatgtctctcatatccctggatctgg 1169
                      * **   **  *   *       * ** * **   * *      * *      **

mouse_Granzyme_C      agaaagaaagaaagaaagaaagcaaaagataaagaacaagggagatcttattgaaatgaa 1245
human_Granzyme_H      ct------------------tgctaa--ataatt-attcatgattttatctgagtagaaa 1208
                                           ** **  ****   *     **  *  * *    *  **

mouse_Granzyme_C      aagggtgtaagagttagaaaaggacacatgtttgtctccgtg--------------cagt 1291
human_Granzyme_H      aagagccaagaactgagaaaaggagacattcttggttctcagtcactgaggagcttctgc 1268
                      *** *   *  * * ********* ****  ***  **   *              * *

mouse_Granzyme_C      catgatggacttcctgaggtctcaggcctccttaaccacagcctccacatcctctacttt 1351
human_Granzyme_H      agtaccagcctttctgctagctgcagccacagcttcaacagctccacctccttgttcttt 1328
                        *    * *** ***    **   *** *     * *****  *  *  * * * ****

mouse_Granzyme_C      cttgatcttcatcccagtttagcttacctca-ggaggtca--gtgtggtaacagtgtcat 1408
human_Granzyme_H      attcttgctttcaccttgttactcagcagcaggggtgtaaatgtgacagtgccatgtcaa 1388
                       **  *  *    **   ***     *  ** **  ** *  ***      *  *****

mouse_Granzyme_C      ttttacccaaaccagaaaaattccttaagt--------aatgtgacgtcaatcagaagca 1460
human_Granzyme_H      cttaaaccaaaccagataattttaataaaccaccttctataatgacacccatcaaaacca 1448
                       ** * ********** ** **   ***          *   ****  * **** ** **

mouse_Granzyme_C      gaggtgcataggttcaaaatgcctgtgagccccctgagcc------------         1500
human_Granzyme_H      gaaggggacaagtctaggaggtctctgagtttactgtacccatccctccttc         1500
                      ** * * * * **  *  * * ** ****    ***  **
```

Supplementary figure 3. Alignment of the mouse granzyme C and human granzyme H promoters. The mouse granzyme C (**ENSMUSG00000079186**) and human granzyme H (**ENSG00000100450**) promoters were downloaded from the Ensembl database and aligned using Clustal Omega (https://www.ebi.ac.uk/Tools/msa/clustalo/). The sequence 1.5kb upstream of the predicted transcriptional start site is shown. The consensus CRE sequence (TGACGTCA) in the mouse gene and the potential DRE half site (TGACG) in the huma gene are highlighted in yellow.
